# What Is the Best Predictor of Mortality in Perforated Peptic Ulcer Disease? A Population-Based, Multivariable Regression Analysis Including Three Clinical Scoring Systems

**DOI:** 10.1007/s11605-014-2485-5

**Published:** 2014-03-08

**Authors:** Kenneth Thorsen, Jon Arne Søreide, Kjetil Søreide

**Affiliations:** 1 Department of Gastrointestinal Surgery, Stavanger University Hospital, PO Box 8100, 4068 Stavanger, Norway; 2 Department of Clinical Medicine, University of Bergen, Bergen, Norway

**Keywords:** Peptic ulcer perforation, Emergency surgery, Mortality, Risk score, Outcome analysis

## Abstract

**Background:**

Mortality rates in perforated peptic ulcer (PPU) have remained unchanged. The aim of this study was to compare known clinical factors and three scoring systems (American Society of Anesthesiologists (ASA), Boey and peptic ulcer perforation (PULP)) in the ability to predict mortality in PPU.

**Material and Methods:**

This is a consecutive, observational cohort study of patients surgically treated for perforated peptic ulcer over a decade (January 2001 through December 2010). Primary outcome was 30-day mortality.

**Results:**

A total of 172 patients were included, of whom 28 (16 %) died within 30 days. Among the factors associated with mortality, the PULP score had an odds ratio (OR) of 18.6 and the ASA score had an OR of 11.6, both with an area under the curve (AUC) of 0.79. The Boey score had an OR of 5.0 and an AUC of 0.75. Hypoalbuminaemia alone (≤37 g/l) achieved an OR of 8.7 and an AUC of 0.78. In multivariable regression, mortality was best predicted by a combination of increasing age, presence of active cancer and delay from admission to surgery of >24 h, together with hypoalbuminaemia, hyperbilirubinaemia and increased creatinine values, for a model AUC of 0.89.

**Conclusion:**

Six clinical factors predicted 30-day mortality better than available risk scores. Hypoalbuminaemia was the strongest single predictor of mortality and may be included for improved risk estimation.

**Electronic supplementary material:**

The online version of this article (doi:10.1007/s11605-014-2485-5) contains supplementary material, which is available to authorized users.

## Introduction

While peptic ulcer disease has decreased in incidence over the past decades, the epidemiological pattern of the complications, including haemorrhage and perforation, have changed little.^1^ Although outcomes from bleeding ulcers have improved with modern endoscopic and interventional radiological strategies,^2^ the outcomes of perforations have remained fairly unchanged.^3^ Even in recent reports, the mortality from perforated peptic ulcer (PPU) remains up to 27 %^4^–^6^ and complications are reported in 20–50 % of the patients.^6^,^7^ A number of scoring systems for outcome prediction have been reported, yet none appear to be superior and most are investigated in isolation.^8^ Among the most frequently used are the American Society of Anesthesiologists (ASA) physical status classification system,^7^ the Boey score^9^ and the more recently introduced peptic ulcer perforation (PULP) score.^5^ However, only the Boey and PULP scores are designed specifically for the prediction of mortality for PPU patients. The Boey score is the most frequently used score, but with varying degree of accuracy.^7^,^10^,^11^ The PULP score appears more accurate, yet it is more complex and has not been validated outside the original cohort. Consequently, contemporary risk prediction in PPU patients is less well investigated with no universally agreed standard, and an optimal way of outcome prediction in this patient group is not known.

Thus, the aim of this study was to compare known risk scores and clinical and laboratory factors for the prediction of 30-day mortality in a consecutive cohort of patients surgically treated for perforated ulcer.

## Material and Methods

The study was approved as a quality control assurance project according to the Regional Ethics Committee (REK Vest # 2011/713). The study was reported to comply with the Strengthening the Reporting of Observational Studies in Epidemiology (STROBE) statement as best applicable.^12^

### Study Population

The Stavanger University Hospital (SUH) serves as the only hospital in the greater Stavanger area and has a catchment area of about 340,000 inhabitants.

All consecutive patients diagnosed with and operated for a perforated gastric or duodenal ulcer admitted between January 2001 and December 2010 were identified from the hospital administrative electronic database using ICD-10 diagnostic codes (K25 and K26) and relevant codes for surgical procedures (i.e. JDA 60 gastroraphy, JDA 61 laparoscopic gastroraphy, JDH 70 duodenoraphy, JDH 71 laparoscopic duodenoraphy).

Patient demographics, including laboratory values and clinical data, were retrieved from hospital records and surgical notes. Five patients with a malignant ulcer, three patients with PPU identified at autopsy, seven patients conservatively treated without operation and two patients operated on suspicion of PPU without finding any perforation were excluded.

### Clinical Diagnosis and Surgical Treatment

Diagnosis was based on clinical symptoms and signs (i.e presence of peritonitis) supported by imaging (mainly with abdominal computed tomography (CT)). A standard predefined set of laboratory values was obtained on admission for all patients with a suspected ‘acute abdomen’, including, among others, haemoglobin, C-reactive protein (CRP), liver enzymes, bilirubin, albumin and creatinine.

The preferred surgical procedure in our institution is an open or laparoscopic primary closure of the perforation by interrupted sutures covered with a pedicled omentoplasty.^3^ In rare cases with no omentum (or falciform ligament) available, primary closure without omentoplasty was done.

### Study Outcomes

The primary endpoint of the study was mortality defined as death within 30 days of surgery.

### Variables and Definitions

Delay of treatment was measured as time from admittance to hospital until the start of surgery.

Sepsis was defined as the presence of two or more of the sepsis criteria (i.e. temperature >38.0, pulse rate >90 beats per minute, respiration rate >20 per minute) and in addition to infection being proved or likely.

Shock on admission was defined as a systolic blood pressure of <100 mmHg and a heart rate of >90 beats per minute.

Complications were graded using the Clavien-Dindo score.^13^ This classification separates complications into five categories: grades 1 and 2 are mild complications that can be medically treated (e.g. pneumonia or urinary tract infections); grade 3 complications require surgical, endoscopic or radiologic intervention; and grade 4 complications are life-threatening complications. Grade 5 is death of a patient during the primary hospital stay.

#### Clinical Risk Scores

The Boey score^9^ was calculated based upon the presence of shock, delay from admission to surgery of >24 h and a high degree of co-morbidity, such as chronic obstructive pulmonary disease, heart failure and active cancer (defined as current cancer under curative treatment or incurable cancer).

The ASA score^14^ was based on the patients’ pre-existing co-morbidity with present clinical condition at admission taken into consideration. Accordingly, any acute deterioration of the patient at admission (e.g. fulfilling the sepsis criteria or the presence of peritonitis or shock) was incorporated in the ASA score evaluation.

The PULP score^5^ was based on age of >65 years, co-morbidity including liver failure, AIDS and active cancer, concomitant use of steroids, shock on admission, time from admission to surgery of >24 h, serum creatinine of >130 (μmol/l) and the above-mentioned ASA score.

The Boey score originally measures delay of >24 h from perforation to surgery, while the PULP score originally measures time from perforation to admission. Since we measured time from admission to surgery, this time delay is used for the calculation of both the Boey score and the PULP score. This means that a delay of >24 h in this study, represents at least 24 h from perforation to surgery.

#### Defining Cut-Offs for Optimal Sensitivity and Specificity

Optimal cut-off for each continuous variable and risk scores were calculated by the receiver operating characteristics (ROC) curve analysis with assessment of the area under the curve (AUC) and its 95 % confidence interval (95 % CI).^15^ An AUC value of >0.8 is considered excellent (i.e. correctly classifies 80 % of, or four out of five, patients), while an AUC of 0.70–0.80 is considered acceptable, and a value of 0.5 equals the flip of a coin.^16^ Sensitivity and specificity with 95 % CI are given for the optimal cut-off value as defined by the ROC analysis, and in addition, the corresponding positive or negative likelihood ratio (LR+ or LR−) is given.

### Statistical Analysis

Data were analysed using the Statistical Package for Social Sciences (v. 21, SPSS Inc.). ROC analysis was performed by MedCalc (v. 12.7.5, http://www.medcalc.org, MedCalc Software, Ostend, Belgium).

A non-parametric distribution of data was assumed, and appropriate statistical tests were used for descriptive data. Chi-square analysis was done for simple associations between relevant dichotomous values.

Optimal cut-off values (based on ROC analyses) were used for dichotomization of the variables for use in the binary regression analyses. Clinical judgement was used for the cut-off for age, where a clinically defined cut-off was set to 60 years.

Logistic binary regression analysis was performed for mortality as the outcome to identify univariate risk factors. Risk is presented as odds ratio (OR) with 95 % CI. Factors with a *p* value of <0.20 in the univariate analyses were included in the multivariable logistic regression model, and all multivariable analyses were adjusted for gender.

The multivariable regression model was built using the forward conditional mode. Included factors were tested both for their continuous values and for the dichotomized variable, where applicable, to test the robustness of the model. For the final multivariable model, the corresponding ‘predicted probability’ value given for each patient was tested by ROC analysis to estimate the performance of the model by the AUC. In addition, the same was run for each of the ASA, PULP and Boey scores in order to compare the accuracy performance across the models. In addition, the Hosmer and Lemeshow goodness-of-fit test was performed for the final multivariable regression model. For internal validation, boostrapping by 1,000 samples was performed on the final multivariable regression model. All tests are two-sided and *p* values of <0.05 were regarded as statistically significant.

## Results

**Fig. 1:**
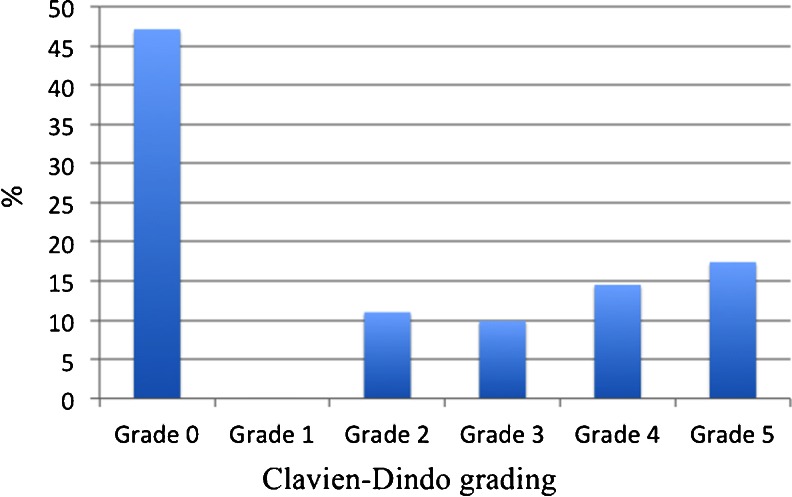
Distribution of complications according to the Clavien-Dindo complication grading systems

The study population comprised 172 patients with a median age of 68 (range 18–101) years. Patient characteristics are given in Table 1. The 30-day mortality was 16.3 % (28/172), and complications were encountered in 52 % (89/172) of the patients (Fig. 1). There were no grade 1 complications recorded. Among the complications recorded were ten suture leakages from the ulcer site, five in the laparotomy and five in the laparoscopy group (*p* = 0.13) Suture leaks were not associated with mortality (*p* = 0.585).

**Table 1:** Clinicopathological characteristics of patients operated for perforated peptic ulcer

Characteristics (*N* = 172)	Age <60 years (*n* = 55)	Age ≥60 years (*n* = 117)	*p* value^a^ for difference
Gender, *n* (%)			
Female	20 (36)	69 (59)	0.006
Male	35 (64)	48 (41)	
Location of ulcer, *n* (%)			
Duodenal	18 (33)	42 (36)	0.606
Gastric	37 (67)	75 (64)	
Delay to surgery (h), median (range)	6.3 (0.5–52.4)	6.2 (1.1–116.2)	0.463
Operation time in min, median (range)	78 (41–210)	79 (34–291)	0.890
Laparoscopy	16 (29)	34 (29)	0.997
Laparotomy	39 (71)	83 (71)	
ASA score			
1–2	47 (85)	52 (44)	<0.001
≥3	8 (15)	65 (56)	
Boey score			
≤1	47 (85)	69 (59)	0.001
>1	8 (15)	48 (41)	
PULP score			
<6	48 (87)	38 (32)	<0.001^b^
≥6	7 (13)	79 (68)	
Median (range)	3 (1–10)	8 (1–14)	
Albumin (g/L)			
Median (range)	42 (20–48)	37 (14–48)	<0.001
Hypoalbuminaemia (≤37 g/l), *n* (%)	9 (5)	63 (37)	<0.001
Bilirubin (μmol/l)			
Median (range)	8 (3–197)	12 (2–481)	0.004
Hyperbilirubinaemia, *n* (%)	6 (4)	18 (11)	0.418
Creatinine (μmol/l)			
Median (range)	78 (27–583)	88 (34–507)	0.124
Creatinine>118	6 (4)	32 (19)	0.015

^a^ *p* value was calculated by chi-square test for categorical data and Mann-Whitney *U* test for continuous data

^b^ *p* value represents differance between PULP scores <6 and ≥6

Optimal cut-off values for each continuous variable based on ROC are given in Table 2. A large variability in discrimination is shown, and none of the variables scored an AUC over 0.8. The LR + was consistently low, with none having a value over 4.

**Table 2:** Optimal cut-off based on receiver operating characteristics (ROC) curve analysis for 30-day mortality

Factor	Cut-off	Sensitivity (95 % CI)	Specificity (95 % CI)	AUC	*p* value of AUC	LR+	LR−
Age (years)	>79	53.6 (33.9–72.5)	83.3 (76.2–89.0)	0.74	<0.001	3.2	0.6
Delay (h)	>14	48.2 (28.7–68.1)	75.5 (67.6–82.3)	0.58	0.22	2.0	0.7
Operation time (min)	≤86	75.0 (55.1–89.3)	41.6 (33.3–50.1)	0.52	0.74	1.3	0.6
ASA score	>3	85.7 (67.3–96.0)	66.0 (57.6–73.7)	0.79	<0.001	2.5	0.2
Boey score	>1	64.3 (44.1–81.4)	94.4 (89.3–97.6)	0.75	<0.001	2.5	0.5
PULP score	>6	92.9 (76.5–99.1)	58.3 (49.8–66.5)	0.79	<0.001	2.3	0.1
Albumin (g/l)	≤37	82.1 (63.1–93.9)	65.5 (57.1–73.3)	0.78	<0.001	2.4	0.3
Bilirubin (μmol/l)	>19	35.7 (18.6–55.9)	90.2 (84.1–94.5)	0.61	0.096	3.7	0.7
Creatinine (μmol/l)	>118	42.9 (24.5–62.8)	81.9 (74.7–87.9)	0.52	0.78	2.4	0.7
CRP (mg/l)	>21	78.6 (59.0–91.7)	52.8 (44.3–61.1)	0.69	<0.001	1.7	0.4

*AUC* area under the curve, *LR*+ positive likelihood ratio, *LR*− negative likelihood ratio, *ASA score* American Society of Anesthesiologists score, *PULP score* peptic ulcer perforation score

### Risk Factors for Mortality

Univariate risk factors associated with mortality are displayed in Table 3. In addition, we analysed for ulcer site, method of operation, presence of cardiovascular disease, prednisolone use, smoking, sepsis, autoimmune disease and NSAID use, but none were statistically significantly associated with mortality. Absence of peritonitis on admission was significantly associated with mortality (*p* = 0.038) by univariate analysis, but not after multivariable regression analysis.

**Table 3:** Univariate regression analysis of factors associated with 30-day mortality

Factors		Deceased	Alive	*p* value	Odds ratio (95 % CI)
Gender	Male	11	72	0.299	0.6 (0.3–1.5)
	Female	17	72		
Age (years)	>60	25	92	<0.001	4.7 (1.4–16.4)
	≤60	3	52		
Delay (h)	>24	9	22	0.027	2.8 (1.1–6.9)
	≤24	18	121		
Preoperative shock	Yes	10	27	0.034	2.6 (1.1–6.2)
	No	17	117		
Active cancer	Yes	9	10	<0.001	6.4 (2.3–17.6)
	No	19	134		
Peritonitis	Yes	13	99	0.034	0.4 (0.2–1.0)
	No	14	44		
ASA score	>3	24	49	<0.001	11.6 (3.8–35.4)
	≤3	4	95		
Boey score	>1	18	38	<0.001	5.0 (2.1–11.8)
	≤1	10	106		
PULP score	>6	26	60	<0.001	18.2 (4.2–79.6)
	≤6	2	84		
Albumin (g/l)	>37	5	93	<0.001	8.7 (3.1–24.4)
	≤37	23	49		
Bilirubin (μmol/l)	>19	10	14	<0.001	5.1 (2.0–13.2)
	≤19	18	129		
Creatinine (μmol/l)	>118	12	26	0.004	3.4 (1.4–8.1)
	≤118	16	118		
CRP (mg/l)	>21	22	68	0.002	4.1 (1.6–10.7)
	≤21	6	76		
Haemoglobin (g/dl)	>12.5	14	106	0.013	2.8 (1.2–6.4)
	≤12.5	14	38		

*ASA score* American Society of Anesthesiologists score, *PULP score* peptic ulcer perforation score

**Fig. 2:**
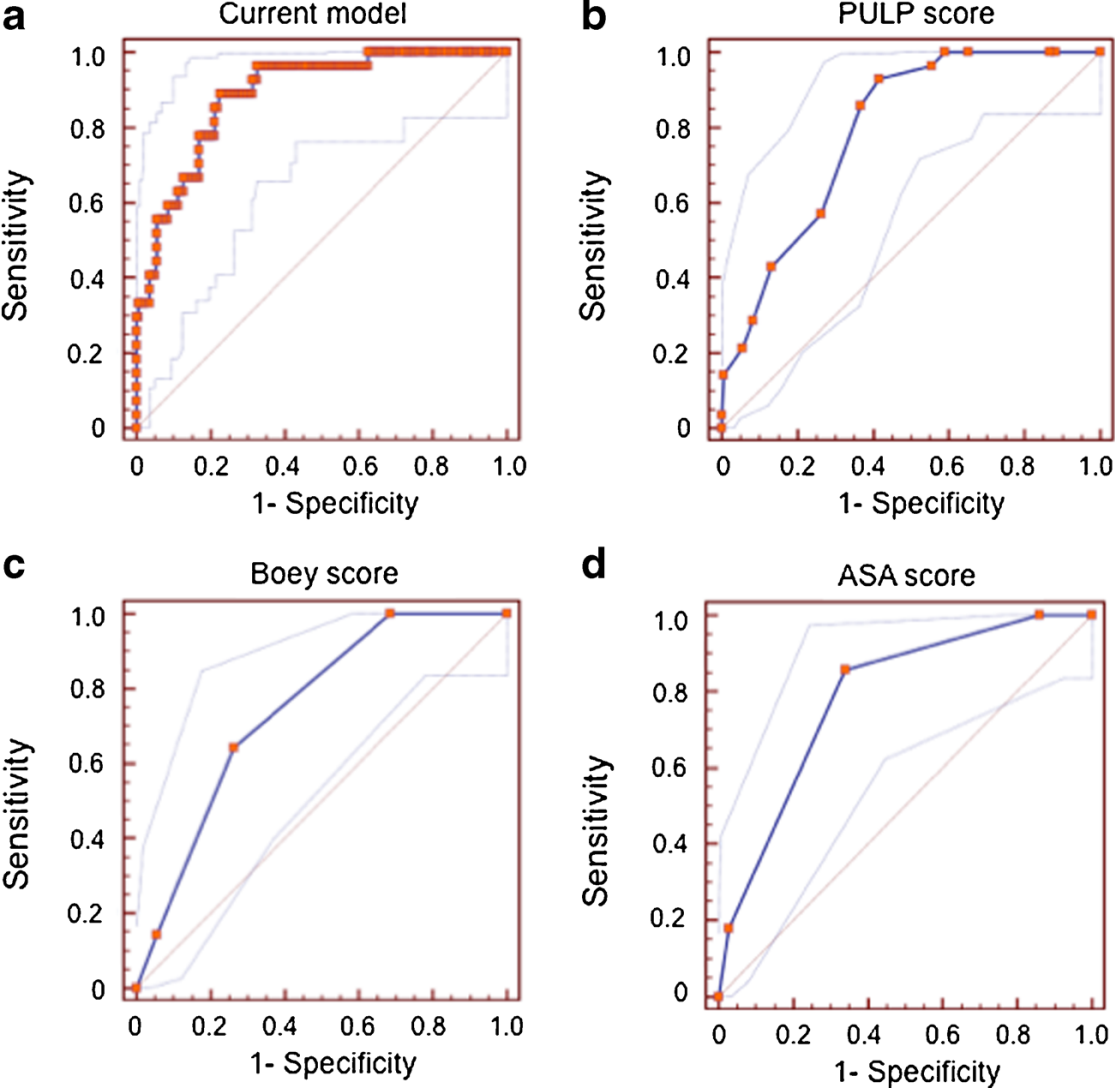
ROC analysis of scores and current model in mortality prediction: **a** current model from the predictive probabilities of the multivariable regression model, **b** the PULP score, **c** the Boey score and **d** the ASA score

During multivariable modelling, non-significant variables were taken out of the regression model for optimization. Adding or leaving the ASA score out of the model only changed the model minimally, and the ASA score was thus left out. The final multivariable regression model for mortality is presented in Table 4. The Hosmer and Lemeshow goodness-of-fit test (*p* = 0.948) indicated a good model fit, and ROC analyses of the predicted probability value gave an AUC of 0.89 (Fig. 2a). The model was internally validated by bootstrapping, changing the *p* values only marginally, thus confirming the validity of the model (see Supplementary info, Table 5).

**Table 4:** Multivariable regression analysis of factors associated with 30-day mortality

Factors	Wald	*p* value	Odds ratio (95 % CI)
Age	10.2	0.001	1.1 (1.0–1.1)
Delay >24 h	4.4	0.035	3.5 (1.1–11.3)
Active cancer	7.8	0.005	7.6 (1.8–31.7)
Albumin ≤37 g/l	5.6	0.018	4.1 (1.3–13.8)
Bilirubin >19 μmol/l	6.5	0.011	5.1 (1.5–18.2)
Creatinine >118 μmol/l	4.4	0.036	3.5 (1.1–11.1)

Adjusted for gender

The predicted probability of each of the clinical scores, as well as the predicted probability used for variables included in the final model (Table 4), is presented for comparison in Fig. 2a–d. The final model had a better AUC and more consistent 95 % CI (Fig. 2a), compared to the PULP (Fig. 2b), Boey (Fig. 2c) and ASA (Fig. 2d) scores.

## Discussion

In the current study, several clinical factors were predictive of 30-day post-operative mortality, of which the combination of increasing age, the presence of active cancer, the state of hypoalbuminaemia, presence of hyperbilirubinaemia, delay to surgery of >24 h and increased creatinine represented the best predictive model. Indicated by an AUC of 0.89, this model would correctly classify nine out of ten patients. Notably, the included factors are all objective measures that are obtainable before surgery and could thus be used for improved risk prediction. While all clinical risk scores evaluated had reasonably accurate ability to predict mortality, none were excellent as deemed by the AUC. Moreover, the single most important factor, the state of hypoalbuminaemia, is not included in any of the three existing risk scores.

An improved risk prediction model may be used for better communication with patients and next-of-kin before surgery for this disease with known high mortality. Obviously, a single predictor cannot be attributed to any individual patient, but the presence of several or all of the most detrimental factors may pose a much greater mortality risk compared to patients with few or none of these attributes. Also, for clinical resource allocation and planning (e.g. risk for prolonged ICU or hospital stay, or need of prolonged care), the combined set of variables may be useful. Finally, the combined score may better allow comparison of patients between studies and allow for case mix adjustments and, importantly, may also allow for potential risk stratification for future clinical trails. Comparison between different patient cohorts from different regions may be valid, as all variables are objective and not influenced by subjective interpretation.

Hypoalbuminaemia was strongly associated with increased mortality, and this is in line with previous reports on perforated peptic ulcer.^17^ Indeed, several past studies found a relation between preoperative hypoalbuminaemia and poor post-operative outcomes across several surgical disciplines.^18^–^20^ This association may be due to the fact that a low serum albumin is closely correlated to a poor preoperative status of the patient, due to chronic disease, presence of underlying cancer, state of cachexia or other causes of malnutrition.

Hyperbilirubinaemia has been found to be associated with perforation in acute appendicitis, and this has been partly explained by a decrease in bile secretion as a consequence of bacteraemia.^21^ Since similar mechanisms may apply for a perforated ulcer, hyperbilirubinaemia may also be of relevance in the management of PPU patients.

Møller et al. found a decrease in mortality from 27 to 17 % after initiating a care bundle protocol.^22^ In the current cohort, the mortality is 16 %, and the two cohorts appear comparable in most aspects. Also, the mortality in our study is in the range of 10–27 % as reported by recent studies from a number of countries, including the USA, Denmark, Scotland, Israel, Nigeria and Ethiopia.^8^,^23^–^28^ As our study includes ‘all comers’ in a defined population with no selection in referral, we believe that the mortality rate is as would be expected for this group of patients.

Most of the deaths in the cohort were attributed to sepsis and multiorgane failure,^6^ which corroborates previous findings in PPU.^29^ However, the presence of sepsis preoperatively was not found to be significantly associated with mortality in the current study. Notably, several other factors may likely be related to the sepsis syndrome and act as surrogates for the presence of sepsis, such as hyperbilirubinaemia and increased creatinine. Increased creatinine levels may be an indicator of several conditions, including chronic renal failure (known or unknown before diagnosis), the expression of pending renal failure (due to the current disease), but may also be due to dehydration or reflect shock or sepsis per se. Nevertheless, increased creatinine is a well-recognized risk factor for mortality both in PPU patients and in other patient groups.^5^,^20^ Indeed, we recognize that several of the factors deemed to be of importance (e.g. albumin, bilirubin and creatinine levels) may be surrogates or indicators for other underlying factors, most likely attributed to pre-existing disease (such as presence of cancer or severe chronic illness) or the state of the acute disease (e.g. reflecting dehydration, state of infection or sepsis, or altered physiology or pending organ failure). We did not attempt to define or investigate causality from the findings in this study. Further investigation into the ‘cause and consequence’ interactions for the better understanding of the nature of this disease is clearly warranted.

Incorporating ‘delay’ as a risk factor is controversial and inconsistent across studies. The Boey score originally measures ‘delay’ as the time from perforation to surgery, which may be prone to error based on recall bias by the patient or record bias when prehospital data are to be obtained in retrospect. On the other side, the PULP score measures ‘delay’ as the time from perforation to admission, thus not including the potential diagnostic delay that may occur in some patients prior to an established diagnosis and start of treatment. In the current study, we have obtained ‘delay’ as the time interval from admission to surgery, as we believe this to be a more robust predictor, as admission to hospital and start of surgery are consistently recorded. Hence, this was applied as the ‘time delay’ variable for both the PULP score and the Boey score. This means that a delay of >24 h (from admission) represents at least 24 h of delay (since symptom debut). Notably, as our institution covers a region with fairly short travelling distances, the time from perforation to admission should not be considerable for most patients. However, we cannot rule out an influence of this on the score parameters. Even though the PULP score achieved both higher OR and AUC values, the Boey score is a much simpler score with higher clinical usefulness than the more complex PULP score, ranging from 0 to 18 points.

The ASA score and the PULP score performed equally well by most aspects in our cohort. The reason for this can to some extent be attributed to our patients receiving higher ASA grading. The ASA grading is known to have interobserver variability, since it is not an objective system.^30^ The ASA grade also varies according to whether or not the acute state is taken into consideration for the ASA grading.^31^ Most of the factors in the PULP score are related to preoperative status and could be included in the ASA score alone, except of age and prednisolone use. Since we included the acute state when grading ASA, it is not so surprising that the PULP score and the ASA score performed equally well. However, the ASA grading which is only based upon pre-existing illness can also be problematic. Clearly, a previous healthy patient presenting with septic shock and acute multiorgane failure is at high risk, but ignoring this in the ASA grading can be misleading.

The question remains how to best improve outcomes for the patients at high risk of dismal outcomes. As recently discussed, there are several important prognostic factors that are unmodifiable, such as age and the presence of active cancer.^3^ However, other important prognostic factors for mortality may be modifiable. As already addressed in the PULP study, the adherence to a sepsis-focused protocol could reduce the mortality by one third compared to conventional treatment,^22^ but when introducing this as a quality-of-care initiative, the factors that improved most were delay to surgery and monitoring of vital parameters, with no significant change in mortality.^32^ To decrease the time interval from perforation to operation appears particularly important as each hour of delay carries with it a worse prognosis.^24^

Some limitations to this study deserve to be mentioned. Although this study involved a consecutive cohort, the data was obtained retrospectively. This may cause difficulty in obtaining accurate data. However, we had little missing data in the variables obtained, likely due to a fairly consistent hospital system with electronic hospital files available for the majority of the study period. A larger study population may have revealed associations not seen in this study; however, most of the results are in line with the recent national data from Denmark,^5^ and as such, this study from the greater Stavanger area should have a wide external validity.

## Conclusion

The combination of age, active cancer, hyperbilirubinaemia, hypoalbuminaemia, elevated creatinine and delay from perforation to surgery of >24 h predicted mortality best.

The new PULP score and the ASA score predicted mortality equally well and better than the Boey score, but none of them were optimal. Hypoalbuminaemia was the strongest single predictor of mortality and may be included for improved risk estimation.

## Electronic supplementary material

Below is the link to the electronic supplementary material.

Supplementary Table 5 (PDF 21 kb)
