# Supplementary material for: What Is the Best Predictor of Mortality in Perforated Peptic Ulcer Disease? A Population-Based, Multivariable Regression Analysis Including Three Clinical Scoring Systems
Source: J Gastrointest Surg. 2014 Mar 8;18(7):1261–8. doi: 10.1007/s11605-014-2485-5 (PMC4057623; doi:10.1007/s11605-014-2485-5)
Supplement: Supplementary file 1 — (PDF 21 kb) [file 11605_2014_2485_MOESM1_ESM.pdf]

Table 5 (supplementary)

Bootstrap for Variables in the Equation

		B	Bootstrap[a]				
			Bias	Std. Error	Sig. (2-tailed)	95% Confidence Interval	
						Lower	Upper
Step 1	albumin37(1)	1.416	.354	1.833	.009	.348	3.522
	bili19(1)	1.637	.162	.782	.011	.447	3.411
	kreat118(1)	1.249	.224	.762	.038	.061	3.024
	Age	.082	.014	.037	.001	.040	.186
	Activecancer(1)	2.034	.282	1.026	.001	.668	4.227
	delay(1)	1.254	.123	.949	.032	-.005	2.960
	Constant	-6.089	-1.127	2.851	.001	-14.201	-3.054

a. Unless otherwise noted, bootstrap results are based on 1000 bootstrap samples
